# Supplementary material for: Fetal MRI reveals altered prenatal cortical surface area in fetuses later diagnosed with autism spectrum disorder
Source: bioRxiv. 2026 Jun 2:2026.06.01.729342. Preprint. [Version 1] doi: 10.64898/2026.06.01.729342 (PMC13252390; doi:10.64898/2026.06.01.729342)
Supplement: Supplement 1 [file NIHPP2026.06.01.729342v1-supplement-1.pdf]

bioRxiv preprint doi: https://doi.org/10.64898/2026.06.01.729342; this version posted June 3, 2026. The copyright holder for this preprint (which was not certified by peer review) is the author/funder, who has granted bioRxiv a license to display the preprint in perpetuity. It is made available under a CC-BY 4.0 International license.

Supplementary Information

7.1. Cohort characteristics

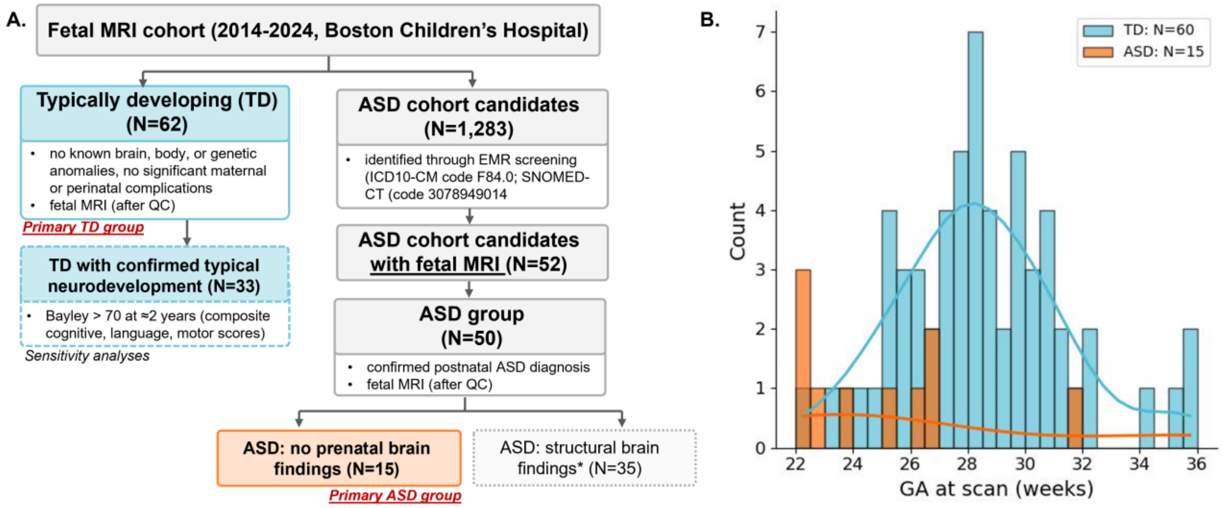

*Most common were changes to ventricular system (83% of subjects) including ventriculomegaly, corpus callosum and other midline anomalies (57%), and other structural dysmorphisms (53%) such as polymicrogyria, heterotopia. Extracerebral brain abnormalities such as cerebellar, vermian and pons hypoplasias (37%), and hemorrhages/acquired injuries (23%) were also relatively common.

C.

subject	Referral for fetal MRI	Known (neurodevelopmental) comorbidities
sub-1	cystic and solid anterior neck mass; suspected inferior cerebellar abnormality	nonverbal learning disorder, intellectual disability, attention-deficit/hyperactivity disorder
sub-2	cystic mass in left lower lung	developmental coordination disorder
sub-3	dilated large bowel; possible anal atresia or bowel obstruction	moderate intellectual disabilities
sub-4	cleft lip	speech sound disorder
sub-5	prominent cavum vergae	attention-deficit/hyperactivity disorder
sub-6	suspected interhemispheric cyst	-
sub-7	fluid collection in right lower abdomen	global developmental delay, attention-deficit/hyperactivity disorder, ASD associated with known genetic condition,
sub-8	renal cyst versus dilated renal pelvis; intrauterine growth restriction	global developmental delay, aggressive behavior, partial idiopathic epilepsy with seizures of localized onset (without status epilepticus)
sub-9	suspected congenital cardiac anomaly; concern for heterotaxy syndrome	-
sub-10	hydrops	attention-deficit/hyperactivity disorder
sub-11	bilateral dilated renal pelves; echogenic bowel	-
sub-12	suspected aortic stenosis, possible Dandy–Walker variant	global developmental delay, nonverbal
sub-13	left lung mass; suspected congenital pulmonary airway malformation	global developmental delay
sub-14	cavum septum pellucidum not visualized on ultrasound	global developmental delay

bioRxiv preprint doi: https://doi.org/10.64898/2026.06.01.729342; this version posted June 3, 2026. The copyright holder for this preprint (which was not certified by peer review) is the author/funder, who has granted bioRxiv a license to display the preprint in perpetuity. It is made available under a CC-BY 4.0 International license.

sub-15	micrognathia	global developmental delay, mixed receptive-expressive language disorder, conductive hearing loss (bilateral)

SI-Figure 1A. Overview of the cohort creation. **B.** GA distribution of the final subject groups. **C.** Prenatal ultrasound findings prompting fetal MRI referral for each included ASD subject and retrospectively identified neurodevelopmental, psychiatric, and related comorbidities documented in the medical record at the time of analysis (May 2026).

7.2. Data quality and processing

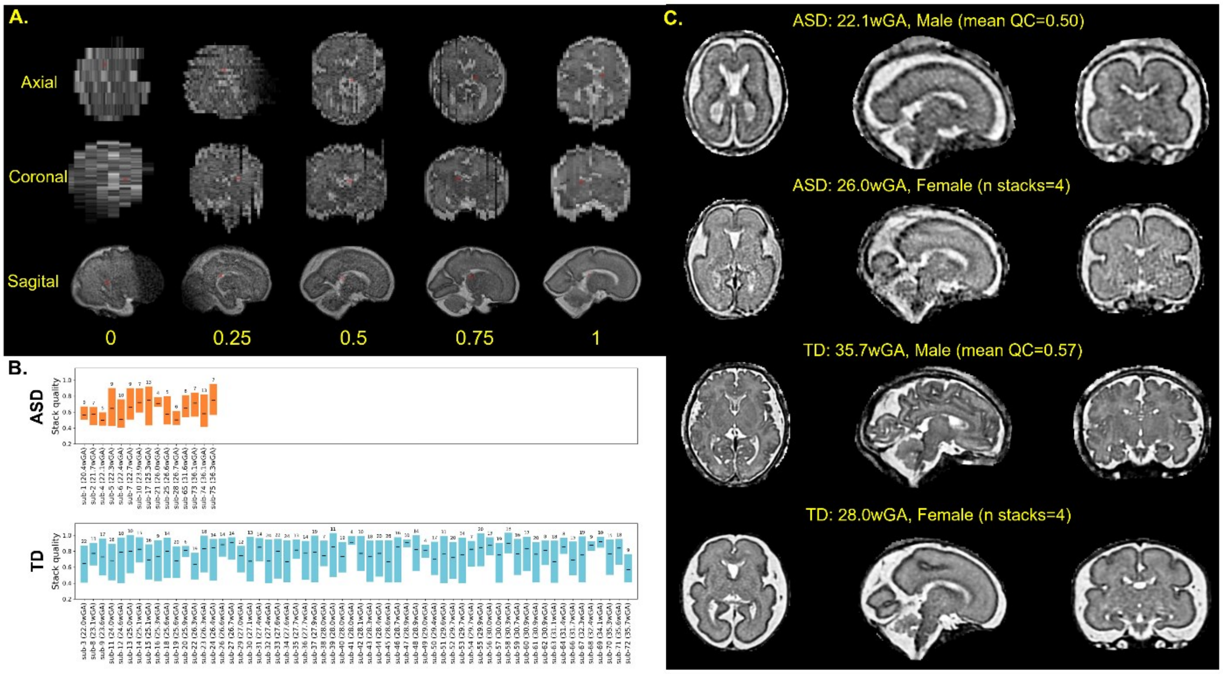

SI-Figure 2A. Illustration of the automated stack quality assessment. Before reconstruction, each available image stack was assigned a value between 0-1 (0: very poor, unusable due to severe motion/blurring; 0.25: poor, usable only for basic reconstruction with evident artifacts; 0.5: acceptable, some blurring but preserved anatomical detail; 0.75: good, minimal blurring, clear tissue boundaries; 1: excellent, sharp anatomical detail, negligible motion artifacts) using a deep-learning model trained on slice-level manual quality ratings from two expert readers as ground truth. Only the subjects with at least 3 stacks with quality ≥0.4 were subsequently reconstructed with NeSVoR (this threshold achieves that stacks with excessive motion and generally low quality do not prevent effective reconstruction). **B.** Distribution of stack quality and numbers across included subjects, ordered by GA at scan. Black bars indicate each subject's mean quality across included stacks; the boxes span the minimum-maximum quality range, and the number above each box shows the final number of stacks used for reconstruction. **C.** Example reconstructions for representative TD and ASD subjects showing subjects with the lowest mean stack quality and subjects with the fewest included stacks.

bioRxiv preprint doi: https://doi.org/10.64898/2026.06.01.729342; this version posted June 3, 2026. The copyright holder for this preprint (which was not certified by peer review) is the author/funder, who has granted bioRxiv a license to display the preprint in perpetuity. It is made available under a CC-BY 4.0 International license.

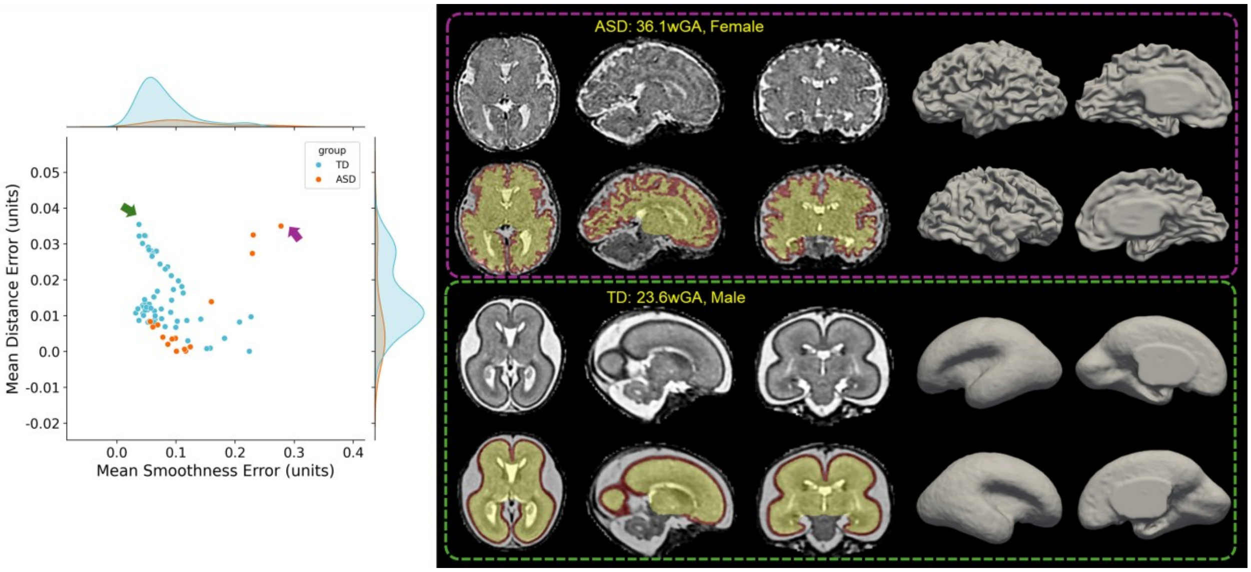

SI-Figure 3. Quality control of surface extraction for the inner CP surface across subjects, together with example T2w reconstruction, their associated tissue segmentation (CP- red, other supratentorial tissue - yellow) in 31w template space for the subjects with the lowest surface extraction quality, as assessed by smoothness (in purple box) and distance errors (in green box).

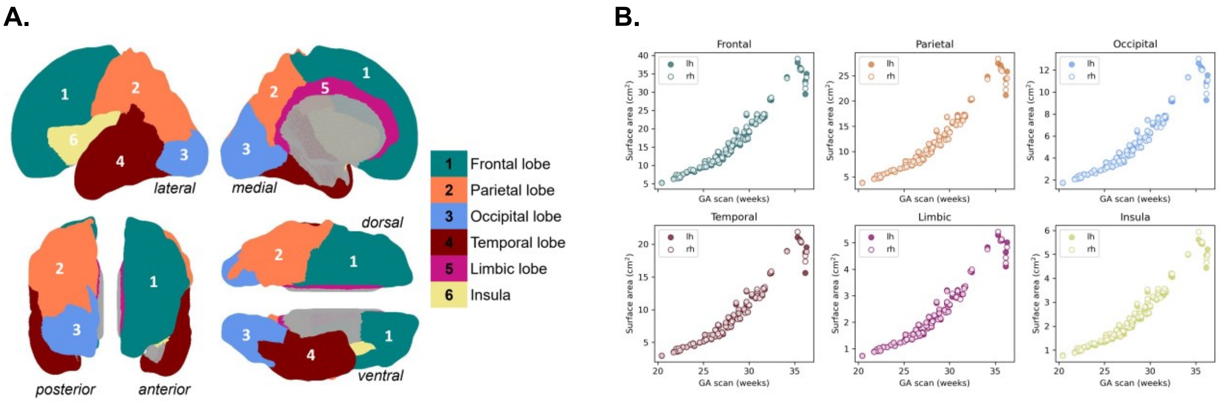

SI-Figure 4. Visualization of the regions for sub-whole-brain level analyses, along with the corresponding color scheme. **B.** Visualization of changes in inner CP surface area with GA showing expected developmental increases.

7.3. Whole-brain analyses

SI-Table 1A. Post hoc pairwise comparisons of estimated marginal means from the linear model (log-transformed whole-brain volume) across group and sex, adjusted for multiple comparisons using the false discovery rate (FDR). **B.** Type II ANOVA results from the linear model of log-transformed CP volume including group, sex, GA (modeled with 3rd order splines), and their interaction. Reported are F-statistics (F), associated p-values (p), and partial eta squared (η²).

**A.**	**Estimate ± SE**	**T (ratio)**	**p**
ASD Female – TD Female	0.02 ± 0.04	0.55	0.812
ASD Female – ASD Male	0.01 ± 0.04	0.34	0.812
ASD Female – TD Male	-0.06 ± 0.04	-1.66	0.205
TD Female – ASD Male	-0.01 ± 0.03	-0.24	0.812

bioRxiv preprint doi: https://doi.org/10.64898/2026.06.01.729342; this version posted June 3, 2026. The copyright holder for this preprint (which was not certified by peer review) is the author/funder, who has granted bioRxiv a license to display the preprint in perpetuity. It is made available under a CC-BY 4.0 International license.

TD Female – TD Male	-0.09 ± 0.02	-4.53	<0.001
ASD Male – TD Male	-0.08 ± 0.03	-2.62	0.033

B.

	**CP V**		
	**F**	**p**	**η2**
group	0.39	0.534	0.00
sex	5.02	0.028	0.07
GA	866.38	<0.001	0.97
group * sex	0.017	0.898	0.00

Sensitivity analyses

1. TD: confirmed typical neurodevelopment subset (N=36)

SI-Table 2A. Type II ANOVA results from the linear model of log-transformed whole volume including group (TD restricted to subjects with postnatal neurodevelopmental follow-up), sex, GA (modeled with 2nd order splines), and their interaction. Reported are F-statistics (F), associated p-values (p), and partial eta squared (η^2). **B.** Same as A but for surface area (GA modeled with 3rd order splines). Note, as group*sex was not significant in primary models, it was not included here.

A.

	**Whole-brain Volume**		
	**F**	**p**	**η2**
group	2.21	0.144	0.05
sex	5.36	0.025	0.11
GA	973.69	<0.001	0.98
group * sex	2.85	0.098	0.06

B.

	**Surface Area**		
	**F**	**p**	**η2**
group	5.57	0.023	0.11
sex	3.70	0.061	0.08
GA	699.12	<0.001	0.98

2. Effect of data quality

SI-Table 3. Group differences in image and surface quality between ASD and TD cohorts. Values are reported as mean [range]. Between-group differences were assessed using independent t-tests with Welch's correction. Reported statistics include t-statistic (t) with corresponding degrees of freedom (dof), p-values (p), and effect sizes (Cohen's d).

	**ASD** *mean [range]*	**TD** *mean [range]*	**t (dof)**	**p**	**Cohen's D**
Mean QC	0.627 [0.50,0.75]	0.780 [0.57,0.92]	6.03 (19.74)	<0.001	1.88
N stacks	7.8 [4,13]	15.3 [4,34]	7.21 (50.68)	<0.001	1.37

a. Using image quality as covariates

SI-Table 4A. Type II ANOVA results from the linear model of log-transformed whole volume including group, sex, GA (modeled with 2nd order splines), their interaction, and additionally measurements of

bioRxiv preprint doi: https://doi.org/10.64898/2026.06.01.729342; this version posted June 3, 2026. The copyright holder for this preprint (which was not certified by peer review) is the author/funder, who has granted bioRxiv a license to display the preprint in perpetuity. It is made available under a CC-BY 4.0 International license.

acquisition quality (mean QC and number of stacks used in reconstruction). Reported are F-statistics (F), associated p-values (p), and partial eta squared (η^2). **B.** Same as A but for surface area (GA modeled with 3rd order splines). Note, as group*sex was not significant in primary models, it was not included here.

A.

	Whole-brain Volume		
	**F**	**p**	**η2**
group	0.13	0.718	0.00
sex	15.36	<0.001	0.19
GA	1346.70	<0.001	0.98
Mean QC	3.54	0.064	0.05
N stacks	3.09	0.083	0.04
group * sex	2.22	0.141	0.03

B.

	Surface Area		
	**F**	**p**	**η2**
group	0.44	0.508	0.00
sex	12.10	<0.001	0.15
GA	1067.49	<0.001	0.98
Mean QC	9.02	0.004	0.12
N stacks	3.62	0.062	0.05

b. Propensity weighting

In attempt to reduce QC imbalance while preserving the full samples size (as opposed to matching TD quality), we also conducted a sensitivity analysis using propensity score weighting. Propensity scores were estimated from mean QC and number of stacks values using generalized linear models (GLMs, *WeightIt* 1.7.0 R package) and used to derive inverse probability weights (weights were trimmed due to limited overlap between ASD and TD groups because of large data quality differences). Weighted linear models were then re-estimated to assess the robustness of group effects after accounting for differences in image quality.

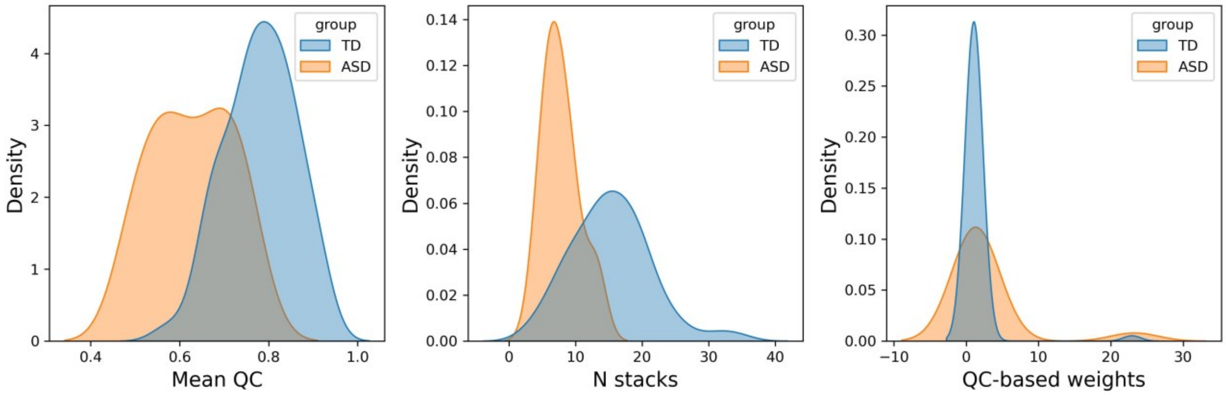

bioRxiv preprint doi: https://doi.org/10.64898/2026.06.01.729342; this version posted June 3, 2026. The copyright holder for this preprint (which was not certified by peer review) is the author/funder, who has granted bioRxiv a license to display the preprint in perpetuity. It is made available under a CC-BY 4.0 International license.

SI-Figure 5. Density plots visualizing group differences in data quality and estimated weights for sensitivity analyses.

SI-Table 5A. Type II ANOVA results from the linear model of log-transformed whole volume including group, sex, GA (modeled with 2nd order splines), and their interaction. The observations were weighted based QC metrics. Reported are F-statistics (F), associated p-values (p), and partial eta squared (η^2). **B.** Same as A but for surface area (GA modeled with 3rd order splines). Note, as group*sex was not significant in primary models, it was not included here.

A.

	**Whole-brain Volume**		
	**F**	**p**	**η2**
group	3.41	0.069	0.05
sex	16.63	<0.001	0.19
GA	1889.18	<0.001	0.98
group * sex	1.74	0.191	0.02

B.

	**Surface Area**		
group	12.49	<0.001	0.15
sex	15.86	<0.001	0.18
GA	2098.55	<0.001	0.98

7.4. Lobar analyses

SI-Table 6. Type III ANOVA results from mixed-effect models of log-transformed lobar surface area (within-subject error, GA 3rd order spline) Reported are F-statistics (F), associated p-values (p), and partial eta squared (η^2).

	**Surface Area**		
	**F**	**p**	**η2**
group	10.11	0.001	0.11
hemi	0.54	0.461	0.03
lobe	-	-	-
sex	10.06	0.002	0.13
GA	3042.00	<0.001	0.98
group*hemi	4.57	0.033	0.01
group*lobe	19.31	0.002	0.08
hemi*lobe	6.16	0.291	0.05
group*hemi*lobe	10.02	0.075	0.01

Sensitivity analyses

1. TD: confirmed typical neurodevelopment subset (N=36)

bioRxiv preprint doi: https://doi.org/10.64898/2026.06.01.729342; this version posted June 3, 2026. The copyright holder for this preprint (which was not certified by peer review) is the author/funder, who has granted bioRxiv a license to display the preprint in perpetuity. It is made available under a CC-BY 4.0 International license.

SI-Table 7. Type III ANOVA results from mixed-effect models of log-transformed lobar surface area (within-subject error, GA 3rd order spline). Reported are F-statistics (F), associated p-values (p), and partial eta squared (η²).

	Surface Area		
	**F**	**p**	**η2**
group	5.72	<0.001	0.10
hemi	0.58	0.017	0.03
lobe	-	-	-
sex	3.62	0.057	0.07
GA	2070.10	<0.001	0.98
group*hemi	3.12	0.077	0.01
group*lobe	15.35	0.009	0.09
hemi*lobe	6.56	0.255	0.06
group*hemi*lobe	7.73	0.172	0.01

2. <u>Effect of data quality</u>
    a. Using image quality as covariates

SI-Table 8A. Type III ANOVA results from mixed-effect models of log-transformed lobar surface area (within-subject error, GA 3rd order spline), including measurements of acquisition quality (mean QC and number of stacks used in reconstruction) as covariates. Reported are F-statistics (F), associated p-values (p), and partial eta squared (η²). **B.** ASD-TD lobar surface area differences derived from A. Group differences were assessed using pairwise contrasts. Reported measures include: estimated marginal means (EMMs; predicted at mean GA=28.1, male sex) and associated 95% confidence intervals (CIs) in the original (back-transformed) scale for interpretability, Wald t-values (t) from log-scale contrasts, associated p-values (p; adjusted for multiple comparisons using FDR method), and effect sizes (d; computed as standardized log-scale mean difference using the standard deviation of log-transformed surface area).

A.

	Surface Area		
	**F**	**p**	**η2**
group	0.39	0.535	0.00
hemi	6.33	0.012	0.08
lobe	-	-	-
sex	12.10	0.001	0.15
GA	3129.60	<0.001	0.98
Mean QC	9.23	0.002	0.12
N stacks	3.66	0.056	0.05
group*hemi	1.58	0.209	0.00
group*lobe	18.35	0.003	0.07
hemi*lobe	15.51	0.008	0.08
group*hemi*lobe	6.86	0.231	0.00

**B**	**ASD [95% CI]**	**TD [95% CI]**	**t**	**p**	**d**

bioRxiv preprint doi: https://doi.org/10.64898/2026.06.01.729342; this version posted June 3, 2026. The copyright holder for this preprint (which was not certified by peer review) is the author/funder, who has granted bioRxiv a license to display the preprint in perpetuity. It is made available under a CC-BY 4.0 International license.

**Left**	***Frontal***	14.57 [13.77–15.42]	14.85 [14.52–15.18]	-0.62	0.537	-0.02
	Parietal	10.58 [10.00–11.20]	10.77 [10.53–11.01]	-0.57	0.574	-0.02
	Occipital	4.79 [4.52–5.07]	4.85 [4.74–4.96]	-0.40	0.693	-0.01
	Temporal	8.14 [7.70–8.62]	8.23 [8.05–8.41]	-0.34	0.734	-0.01
	Limbic	2.04 [1.93–2.16]	2.06 [2.01–2.10]	-0.19	0.850	-0.01
	Insula	2.14 [2.02–2.26]	2.19 [2.14–2.24]	-0.74	0.462	-0.03
**Right**	***Frontal***	14.71 [13.90–15.57]	15.07 [14.74–15.41]	-0.81	0.422	-0.03
	Parietal	10.59 [10.01–11.21]	10.73 [10.49–10.97]	-0.43	0.671	-0.01
	Occipital	4.81 [4.55–5.09]	4.84 [4.73–4.95]	-0.18	0.857	-0.01
	Temporal	8.23 [7.78–8.71]	8.29 [8.11–8.47]	-0.22	0.824	-0.01
	Limbic	2.05 [1.94–2.17]	2.06 [2.02–2.11]	-0.15	0.880	-0.01
	Insula	2.18 [2.06–2.31]	2.24 [2.19–2.29]	-0.94	0.352	-0.03

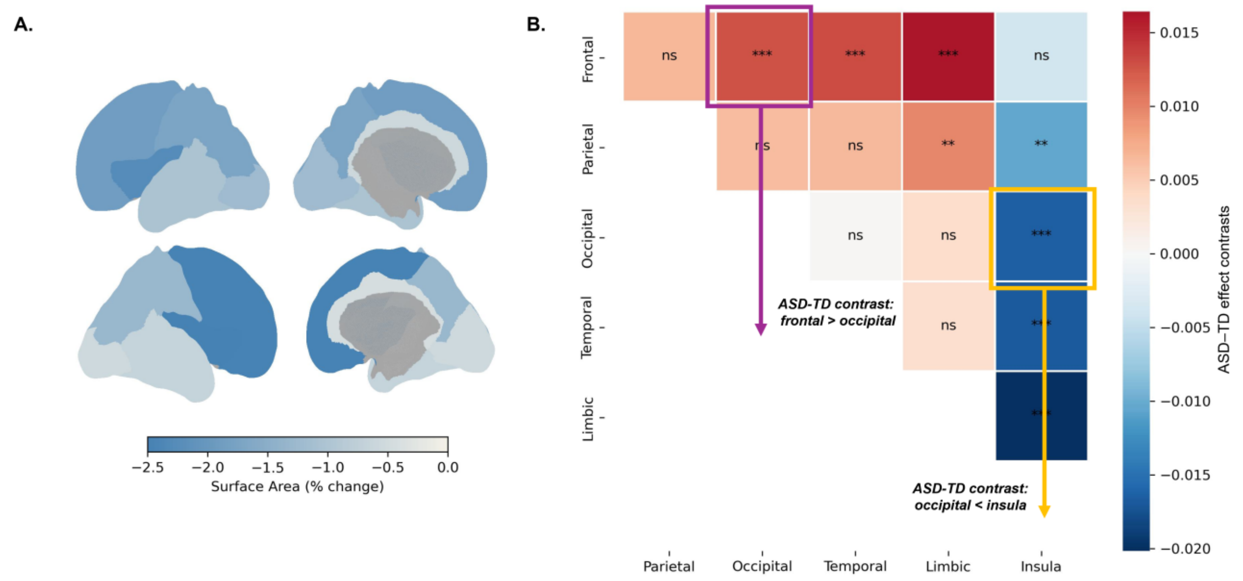

SI-Figure 6A. Relative ASD-TD differences summarizing results in SI-Table 9 (sensitivity analysis including acquisition quality mean QC and number of stacks as covariates). No relative changes are significant after accounting for data quality differences; the overall pattern of surface area reductions in ASD is maintained. **B.** Spatial heterogeneity in group effects from pairwise comparisons. Some lobes have significantly more pronounced surface area reductions in ASD-TD than others (row-column direction, i.e. red in row means given lobe is shows higher reductions than its pair in column, blue means less reduction). Significance codes: p<0.001 (***), p<0.01 (**), p<0.5 (*), p>0.5 (ns); FDR corrected.

A.

	Left		Right	
	**t**	**p**	**t**	**p**
Frontal	-0.95	0.888	-1.45	0.888
Parietal	-0.68	0.888	-0.95	0.888
Occipital	-0.27	0.888	-0.26	0.888

B.

bioRxiv preprint doi: https://doi.org/10.64898/2026.06.01.729342; this version posted June 3, 2026. The copyright holder for this preprint (which was not certified by peer review) is the author/funder, who has granted bioRxiv a license to display the preprint in perpetuity. It is made available under a CC-BY 4.0 International license.

Temporal	-0.52	0.888	-0.54	0.888
Limbic	-0.03	0.976	-0.24	0.888
Insula	-0.65	0.888	-1.52	0.888

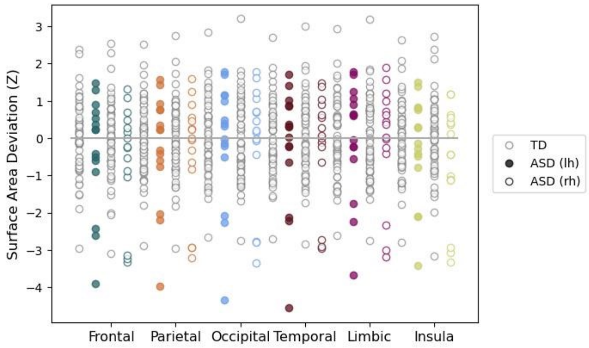

SI-Figure 7A. ASD normative deviations in lobar surface area (z-scores relative to TD normative model). Statistical significance of deviation from zero was assessed using mixed-effects estimated marginal means with FDR correction. Negative values indicate reduced surface area relative to TD expectations. (As z-scores are standardized relative to the TD normative model, mean Z values reflect effect sizes in units of TD standard deviations.) **B.** Scatter plots of subject-level deviation scores.
